# Distance between the center of the FAZ measured automatically and the highest foveal bulge using OCT-angiography in elderly healthy eyes

**DOI:** 10.1038/s41598-021-00826-1

**Published:** 2021-11-02

**Authors:** Takuhei Shoji, Hirokazu Ishii, Junji Kanno, Takanori Sasaki, Yuji Yoshikawa, Hisashi Ibuki, Kei Shinoda

**Affiliations:** Department of Ophthalmology, Saitama Medical University, 38 Morohongo Moroyama-machi, Iruma, Saitama 350-0495 Japan

**Keywords:** Anatomy, Medical imaging

## Abstract

The center of the fovea, termed the foveola, is the area of highest visual acuity, has the highest density of cone photoreceptors. We investigated the distance between the automatically-determined center of the foveal avascular zone (FAZ) and the manually-determined highest foveal bulge (FB) point using single swept-source optical coherence tomography angiography (OCTA) instrument. This cross-sectional study included 49 eyes of 49 individuals (34 women and 15 men; median age: 68 years) with no history of ocular disorders. The FAZ in the superficial capillary plexus was automatically determined using the Kanno–Saitama macro method, and the center of the FAZ was automatically determined using ellipse approximation. Another candidate foveal center, the highest FB point, was determined manually on the serial cross-sectional B-scan images. As a result, the foveal center was manually identified as the highest FB point on B-scan OCTA images. The center of the FAZ was more likely to be located inferior to the highest FB point (*p* = 0.031). In participants with a total (linear) distance of more than 50 μm between the center of the FAZ and the highest FB point, the displacement was significantly more in the horizontal direction than in the vertical direction (*p* = 0.017). These results can be applicable to further studies regarding the spatial relationships between the center of the FAZ and the highest FB point in various macular diseases or previously-treated eyes.

## Introduction

The center of the fovea, termed the foveola, is the area of highest visual acuity, has the highest density of cone photoreceptors and the smallest thickness of the fovea, and is the center of the foveal avascular zone (FAZ)^1,2^. Optical coherence tomography (OCT) is a non-invasive imaging technology used to acquire high-resolution, 3D, cross-sectional images of the retina, and is one of the most important ancillary tools for the diagnosis and management of macular diseases^3,4^. Both spectral-domain OCT (SD-OCT) and swept-source OCT (SS-OCT) provide detailed and in vivo analyses of the interior of the retina, especially the fovea, which is the area with the highest visual acuity. Therefore, the accurate identification of the foveal center is of great significance for disease evaluation and diagnosis.

Previous studies of B scan images of healthy eyes have reported that the ellipsoid zone (EZ) has a bulge at the central fovea^5,6^. This point is defined as the foveal bulge (FB) and is regarded as the center of the fovea^5–7^. The FB is observed on cross sectional B scan image using OCT or OCT-angiography (OCTA) instruments. The presence or absence of the FB has also been shown to be significantly correlated with visual acuity in various retinal disorders such as branch retinal vein occlusion, macular dystrophy, amblyopia, and eyes after rhegmatogenous retinal detachment repair^5,8,9^.

The perifoveal vascular network is composed of interconnected capillaries that perfuse the inner retinal layer. This network forms a ring at the margin of the fovea and produces a capillary-free region referred to as the FAZ^10^. The FAZ is highly sensitive to ischemic events and has been implicated in several pathological processes. Previous studies have reported enlarged FAZs in retinal ischemic diseases (including diabetic retinopathy)^11^ and retinal vascular obstruction^12^. In addition, the FAZ has been evaluated in glaucoma^13–15^, and idiopathic epiretinal membrane (ERM)^16^. In recent reports, the center of foveal pit (FP), center of the FB, and center of the FAZ have been located manually using OCT angiography (OCTA), though the manually-located landmarks are not always reproducible^16,17^. Recently, we generated and reported a macro program to automatically determine the FAZ area using OCTA^15,18^.

Although previous studies have suggested that both the FAZ and the highest FB point are critical biomarkers of the functional properties of the fovea in eyes with various retinal diseases, the spatial relationship between the center of the FAZ in en-face images and the highest FB point are poorly understood. Therefore, this study investigated the positional relationship between en face OCTA-derived center of FAZ using an automated FAZ extraction program, and the manually-determined highest FB point, which is determined as in the previously reported method, using cross-sectional B scan images obtained from the single OCTA system in elderly healthy eyes.

## Methods

### Study population

This cross-sectional study of healthy subjects was approved by the Ethics Committee of Saitama Medical University (No. 20013.01) and was conducted in accordance with the tenets of the Declaration of Helsinki. Healthy participants that were 20 years of age or older who fulfilled the eligibility requirements were enrolled in the study. All participants provided informed consent. The study period was October 2017 to November 2017.

Healthy participants were recruited from the ophthalmology outpatient clinic of Saitama Medical University Hospital (Saitama, Japan). All participants underwent a comprehensive ophthalmic examination, including slit lamp biomicroscopy, intraocular pressure (IOP) measurement via non-contact tonometry (Tonoref II, Nidek Co., Ltd., Aichi, Japan), and fundus photography (CX-1, Canon Inc., Tokyo, Japan). The axial length and central corneal thickness (CCT) were measured using an optical biometer (OA-2000, Tomey Corp., Nagoya, Japan). Horizontal and vertical B-scan images and en-face images of the area around the macula were obtained using swept-source optical coherence tomography angiography (SS-OCTA; PlexElite9000 Carl Zeiss Meditec, Jena, Germany).

Participants < 20 years of age with a reflective error > + 3.0 diopters or < − 6.0 diopters, an axial length > 26 mm, other ocular diseases, diabetic retinopathy, retinal vein/artery occlusion, age-related macular degeneration, retinal detachment, tilted disc syndrome, exfoliation syndrome, high myopia, or a history of ocular surgery (except uncomplicated cataract surgery) were excluded from the study. Participants in whom images of poor quality were obtained (signal strength < 8 due to signal noise; 1 = minimum, 10 = maximum) were also excluded from the study.

### Optical coherence tomography angiography

A 3 × 3 mm (1024 × 1024 pixels) OCTA image centered on the fovea was scanned using SS-OCTA using a central wavelength of 1060 nm, an A-scan rate of 100,000 scans per second, an axial resolution of approximately 5 μm in tissue, and an estimated lateral resolution at the retinal surface of approximately 12 μm^19^. The angiography image was processed using phase/Doppler shift and amplitude variation (Optical Micro-Angiography)^20^. The scan contained 300 A-lines × 300 locations with four repeated scans in each fixed location.

### Measurements of the center of the FAZ using OCTA en-face images

**Figure 1:**
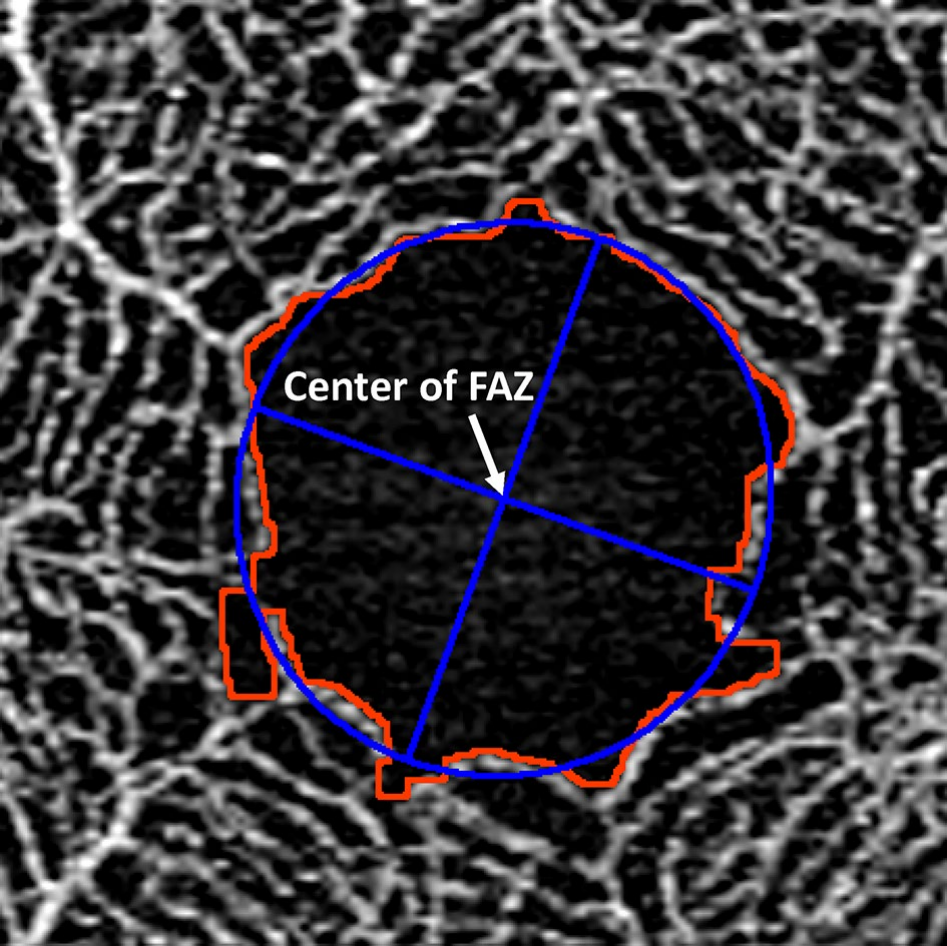
Identification of the center of the foveal avascular zone. The central fossa vessel-free zone was extracted using the Kanno–Saitama Macro (KSM) method (red line). An approximate ellipse was created for the delineated foveal avascular zone (FAZ) (blue line). The center of the FAZ is defined as the intersection of the major and minor axes of the approximate ellipse. This figure was created using SS-OCTA (PlexElite9000 Carl Zeiss Meditec, Jena, Germany) and ImageJ software.

The area of the FAZ (mm^2^) was calculated using ImageJ (National Institutes of Health, Bethesda, Maryland, USA) and an original macro language (KSM program), as previously described^18^. In brief, the SS-OCTA software generates en-face images from slabs at different layers via automated segmentation. The superficial retinal layer (SRL) was used to measure the area of the FAZ. The SRL was defined as the area between the internal limiting membrane layer and the inner plexiform layer. KSM is an automated analysis program that extracts the area of the FAZ. The FAZ was defined as the area denoted by the connected points along the borders of the identifiable capillary network in the parafoveal area. The extracted FAZ showed excellent reproducibility and was comparable to manual measurements^18^. After identifying the FAZ, the center of the FAZ was automatically identified as the center of an elliptic approximation using the ImageJ program (Fig. 1).

### Measurements of the height of the FB

**Figure 2:**
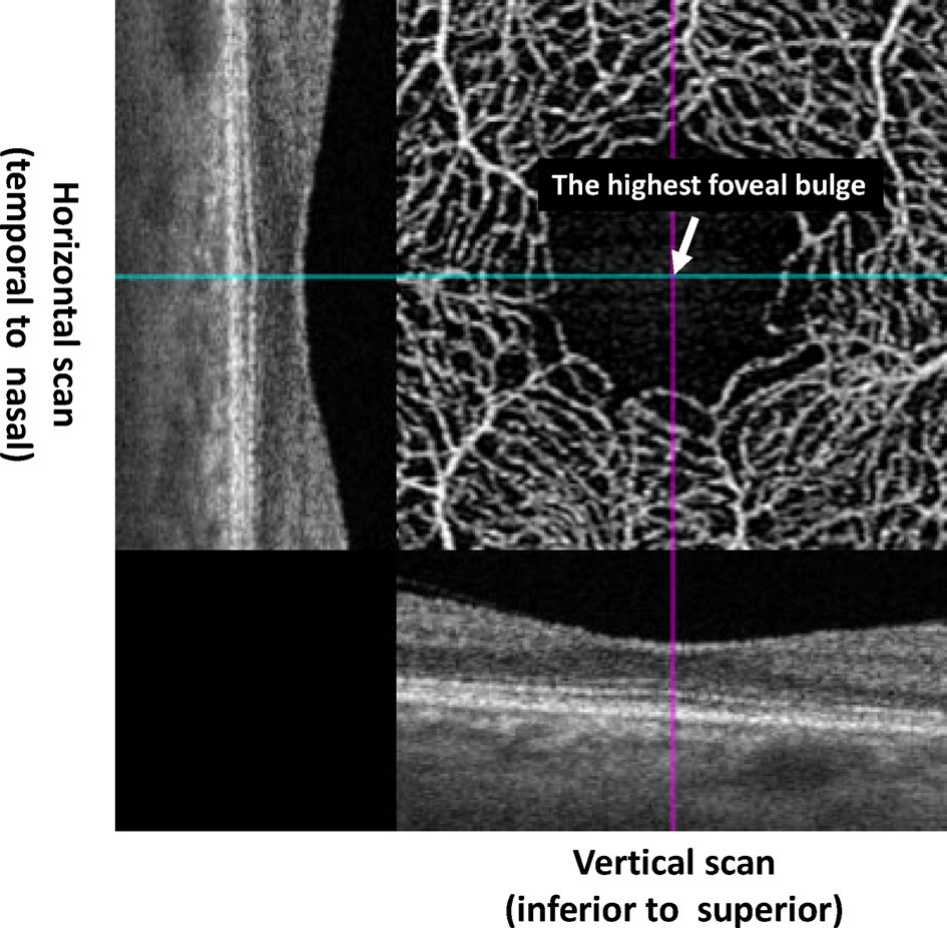
Identification of the highest foveal bulge point using optical coherence tomography angiography. The intersection of the highest foveal bulge (FB) in the horizontal and vertical scans was defined as the highest FB point. This figure was created using SS-OCTA (PlexElite9000 Carl Zeiss Meditec, Jena, Germany) and ImageJ software.

**Figure 3:**
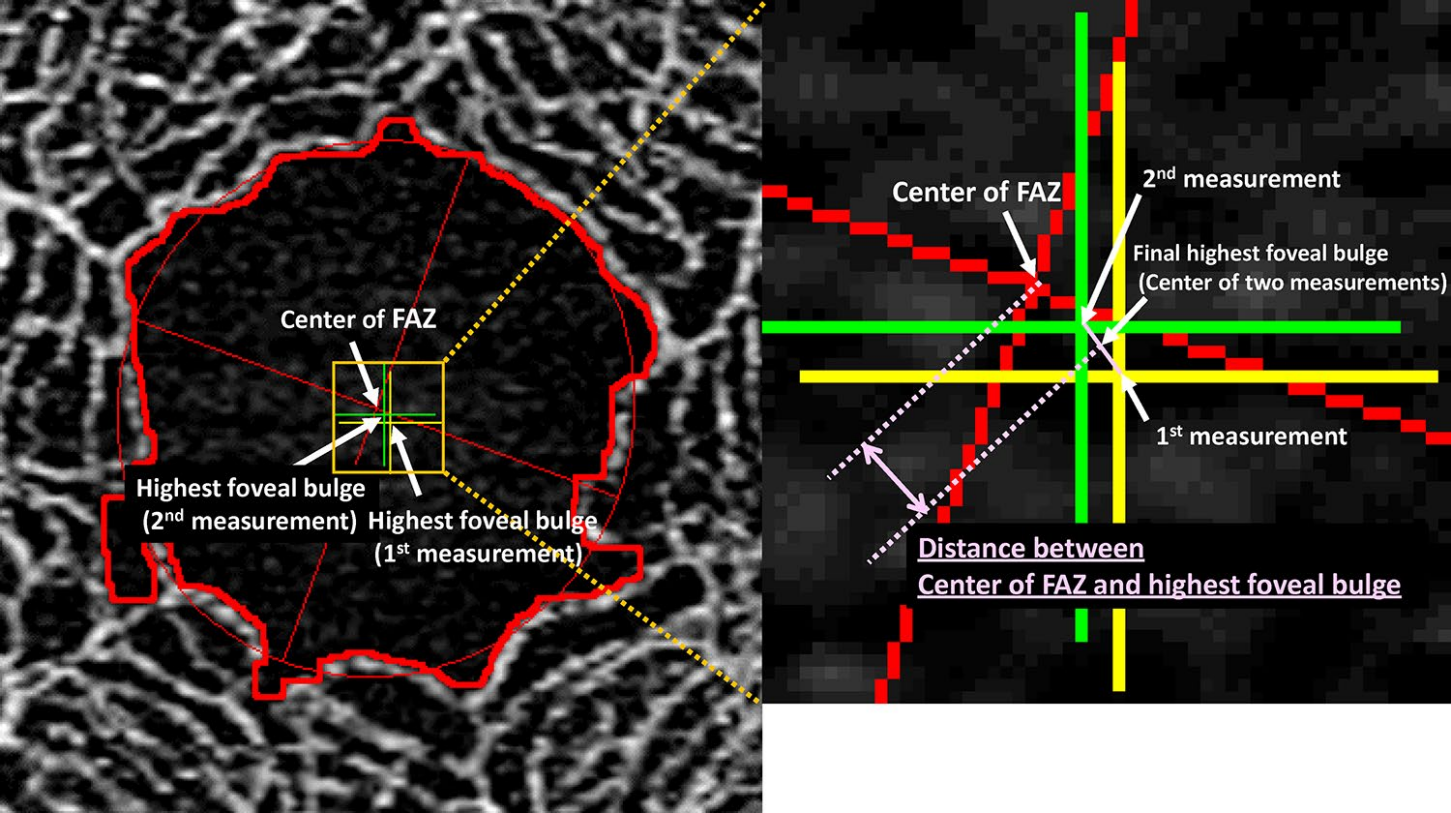
Schematic explanation of the distance between the center of the foveal avascular zone and the highest foveal bulge point. The red cross shows the center of the foveal avascular zone (FAZ) on the optical coherence tomography angiography (OCTA) image. The green and yellow crosses show the highest foveal bulge (FB) points that were measured manually using a B-scan image. The final highest FB point is defined as the center of the two measured highest FB points. The distance between the center of the FAZ and the highest FB point is measured (pink arrow). This figure was created using SS-OCTA (PlexElite9000 Carl Zeiss Meditec, Jena, Germany) and ImageJ software.

The height of the FB point was measured at the level of the thickest FB point, as previously reported^16,17^. The height of the FB was defined as the distance between the outer boundary of the ellipsoid zone (EZ) and the inner boundary of the Bruch’s membrane at the vertex of the FB (Fig. 2). A single observer (HI) who was blinded to the participants’ characteristics measured the highest FB points in both the horizontal and vertical B scans in duplicate. The intersection of the highest FBs in the horizontal and vertical scans was defined as the highest FB point. The distance between the central point of the FAZ and the highest FB point was calculated (Fig. 3). The total (linear) distance was calculated as the square root of the sum of the squares of the horizontal and vertical distance. The magnification effect was adjusted due to axial length according to the manufacturer's correction formula, and it was confirmed that the correction was comparable to that in a previous study^21^.

### Statistical analysis

The distribution of numerical variables was assessed via the inspection of histograms and the use of the Shapiro–Wilk W test for normality. Normally-distributed variables are reported as mean ± standard deviation. Non-normally distributed variables are reported as median (IQR). The center of the FAZ from horizontal and vertical positions of the highest FB points were determined using a one-sample Wilcoxon signed rank test with ± 0 as the reference point. The Wilcoxon signed-rank test was used to compare the horizontal and vertical distances. Statistical significance was set at *p* < 0.05. All statistical analyses were conducted using JMP version 10.1 software (SAS Institute Inc., Cary, NC, USA) and Stata software version 15 (StataCorp LP, College Station, TX, USA).

## Results

**Figure 4:**
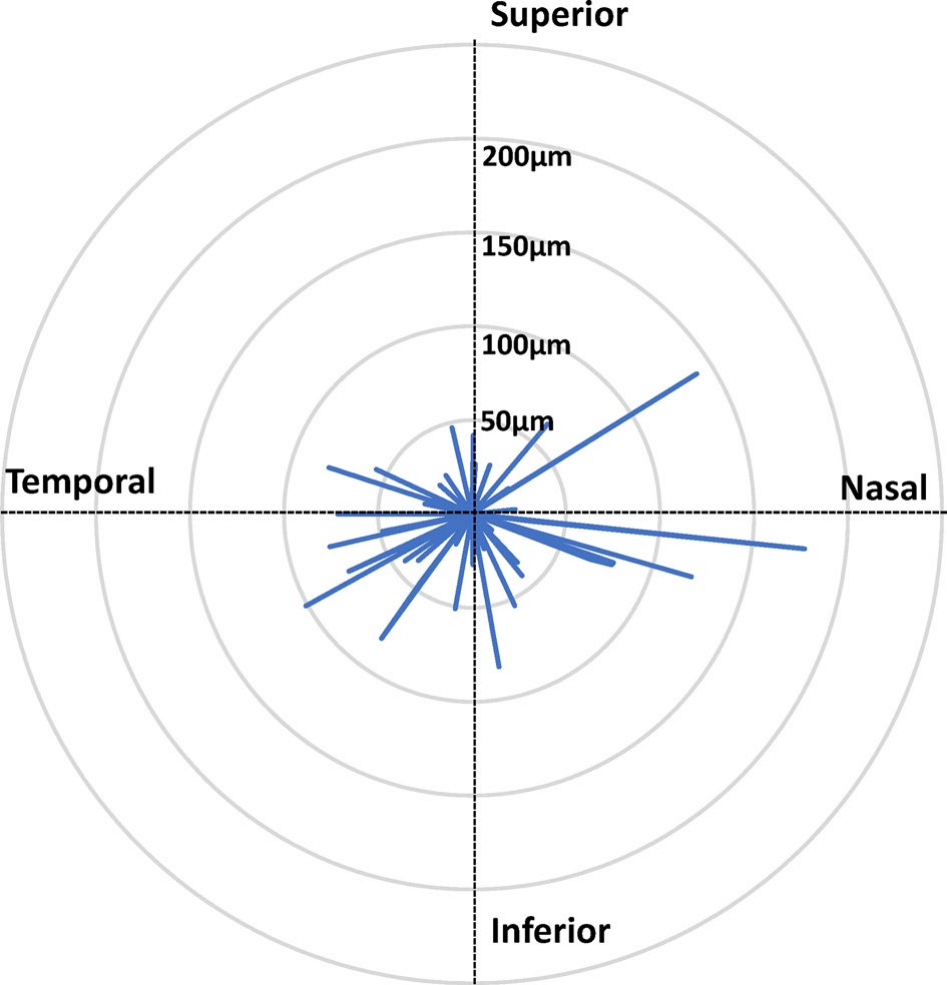
Direction and distance of the eccentric position of the center of the foveal avascular zone based on highest foveal bulge point. Each line indicates the distance and direction between two points in each eye. If the line points nasally and superior, it means that the center of the FAZ was located nasally and upward relative to the highest foveal bulge point. This figure was created using ImageJ software.

**Figure 5:**
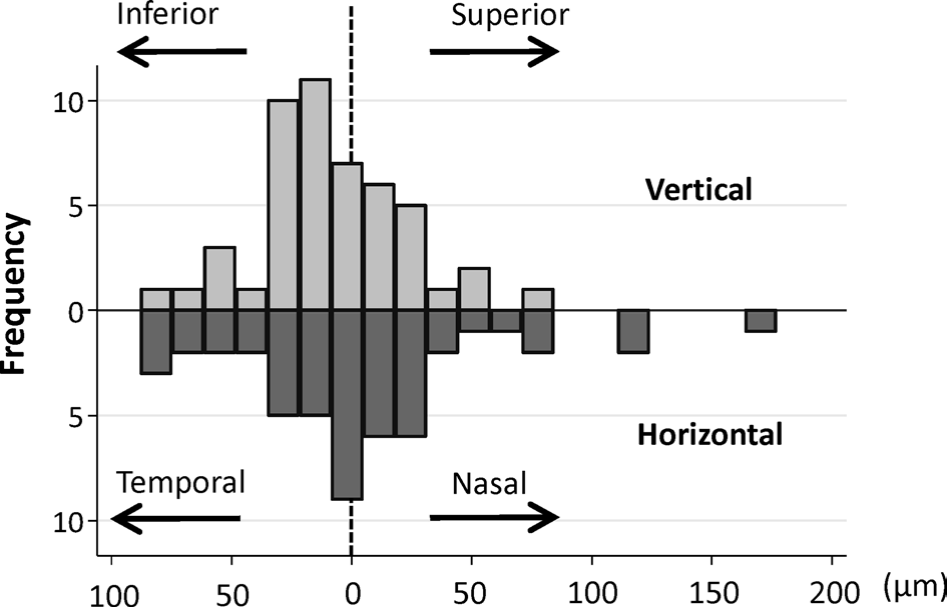
Distribution of the distance from the highest foveal bulge point to the center of the foveal avascular zone in the vertical and horizontal directions. Eye distribution of the distance from the highest foveal bulge (FB) point to the center of the foveal avascular zone (FAZ) in the vertical (top row) and horizontal (bottom row) directions. The peak distribution in the vertical direction is biased inferior. This means that many eyes had the center of the FAZ located inferior side than the highest FB point. This figure was created using Stata software version 15 (StataCorp LP, College Station, TX, USA).

This study included 49 eyes from 49 healthy participants. The participants’ ocular characteristics are presented in Table 1. The median participant age was 68 years (interquartile range (IQR): 67–72 years), and 34 participants (69.4%) were women. The decimal best-corrected visual acuity (BCVA) values were converted to logarithm of the minimum angle of resolution (logMAR) values. The median logMAR was − 0.08 (IQR: − 0.08 to − 0.08), axial length was 23.3 mm (IQR: 22.8–23.9 mm), spherical equivalent was − 0.00 diopters (IQR: − 0.38 to 1.25 diopters), and IOP was 14.3 mmHg (IQR: 12.3–16.3 mmHg). Figure 4 shows the direction and distance of the eccentric position of the center of the FAZ from the highest FB point. Each line indicates the distance and direction between two points in each eye. If the line points nasally and superior, it means that the center of the FAZ was located nasally and upward relative to the highest FB point. Figure 5 shows the distribution of the distance from the highest FB point to the center of the FAZ in the vertical (upper panel) and horizontal directions (lower panel). The distribution of the distance in the vertical direction is mostly inferior to the highest FB point, while the distribution in the horizontal direction is close to the highest FB point. Table 2 shows the positional location of the FAZ from the highest FB point. In the horizontal direction, the number of participants with a nasal- or temporal-shifted center of the FAZ were 26 and 23, respectively. Overall, the median horizontal shift of the center of the FAZ was 2.0 μm in the nasal direction from the highest FB point, which was not statistically significant. In the vertical direction, the center of the FAZ was more likely to be located inferior to the highest FB point (32 of 49 eyes, 65.3%), with a median distance of 10.5 μm from the highest FB point, which was significantly different from the reference value of ± 0 (*p* = 0.031, Wilcoxon signed-rank test).

**Table 1:** Participant characteristics.

No. of patients (n)	49
Age (years)	68 (67, 72)
Gender (male/female)	15/34
BCVA (Log MAR)	− 0.08 (− 0.08, − 0.08)
SE (Diopters)	0.00 (− 0.38, 1.25)
Axial Length (mm)	23.3 (22.8, 23.9)
IOP (mmHg)	14.3 (12.3, 16.3)

Data are presented as numbers or median (interquartile range).

LogMAR represents the log of the decimal best corrected visual acuity.

*LogMAR* logarithm of the minimum angle of resolution, *SE* spherical equivalent, *IOP* intra ocular pressure.

**Table 2:** Distance to the positional location of the foveal avascular zone from the highest foveal bulge point.

	Direction and distance	*p*
**Horizontal direction**		
Nasal/temporal (n)	26/23	
Distance (μm)	2.0 (− 26.2, 23.5) (nasal)	0.914
**Vertical direction**		
Superior/inferior (n)	17/32	
Distance (μm)	10.5 (− 9.0, 26.3) (inferior)	0.031

Data are presented as numbers or median (interquartile range).

*p values obtained using a one-sample Wilcoxson signed rank test with a reference value of zero.

**Figure 6:**
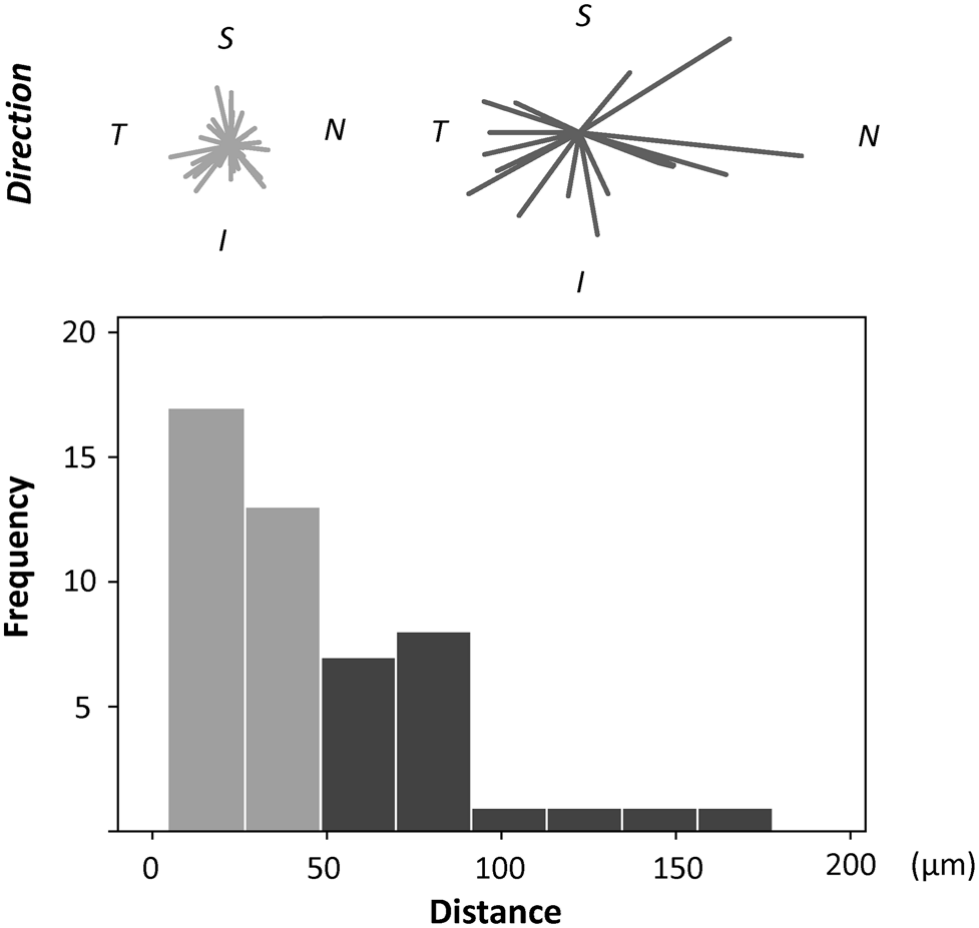
Total distance and direction of the highest foveal bulge point in relation to the center of the foveal avascular zone. The total (linear) distance was divided into two groups: less than 50 μm (light gray lines and bars) and greater than 50 μm (dark gray lines and bars). In the top row, the deviation of each eye in each group is indicated by the direction and length of the line. The bottom row shows the distribution of the total distance between two points. The distribution is shown to be skewed less than 50 μm. This figure was created using Stata software version 15 (StataCorp LP, College Station, TX, USA) and ImageJ software.

Figure 6 shows the direction and distribution of the absolute value of the total (means linear) distance from the highest FB point to the center of the FAZ. Table 3 shows the absolute values of the total, horizontal, and vertical distances and compares the absolute values of the horizontal and vertical distances. In 31 eyes (63.2%), the total distance was less than 50 μm. In these eyes, there was no significant difference in the absolute distance of horizontal direction and vertical direction (*p* = 0.931). In the eyes with a total distance of equal or more than 50 μm (n = 18), the absolute distance was significantly greater in the horizontal direction (median value of 68.5 μm) than in the vertical direction (median value of 28.5 μm) (*p* = 0.017, Wilcoxon signed-rank test).

**Table 3:** Total distance between the center of the FAZ and the highest point of the foveal bulge.

Distance	n	Total distance (μm)	Horizontal (μm)	Vertical (μm)	p*
**Overall**	49	39.7 (23.4–69.5)	24.0 (9.8–53.0)	21.5 (9.5–31.5)	0.053
< 50 μm	31	24.8 (18.1–37.9)	14.0 (4.5–26.5)	14.5 (6.5–26.0)	0.931
≧ 50 μm	18	78.3 (61.6–87.3)	68.5 (46.5–79.0)	28.5 (23.0–49.8)	0.014

Data are presented as numbers or median (interquartile range).

*p values were calculated by comparing the absolute values of horizontal and vertical distances using the Wilcoxon signed-rank test.

## Discussion

This study evaluated the spatial relationship between the automatically-determined center of the OCTA-derived FAZ and the center of the highest FB point. The distance between the center of the FAZ and the highest FB point was generally close but was not aligned in healthy eyes, with an average distance of approximately 40 μm between the two points. While the positions of the center of the FAZ and the highest FB point were similar in the horizontal direction, the center of the FAZ was located significantly inferior to the highest FB point in the vertical direction. In participants with a total distance of more than 50 μm between the center of the FAZ and the highest FB point, the displacement was significantly more in the horizontal direction than in the vertical direction.

In this study, the FAZ was identified automatically using the Kanno–Saitama Macro (KSM) method^18^. Unlike our study, manual segmentation of FAZ was required when using OCTA devices programs in previous studies^16^. The automated identification of the FAZ has excellent reproducibility, is comparable to measurements obtained manually, and the specific KSM method and its clinical applications have been reported^15,18^. The center of the FAZ and the highest FB point is clinically relevant as the macular region, including the fovea, is closely associated with visual prognosis.

Prior to the widespread use of OCTA, the positional relationship between the center of the FAZ and the highest FB point was poorly understood as FAZ measurements using fluorescein angiography were not routine, especially in healthy individuals. Furthermore, the ability to accurately compare and measure the distance between the center of the FAZ and the highest FB point was made possible with the development of OCTA technology, which provides both in vivo en-face images and B scan images of the macular region as digital images using a single instrument.

Several recent studies have reported that even in healthy eyes, the highest FB point, FP, and the center of FAZ, which are considered to indicate the center of fovea, are misaligned^16,17,22^. Regarding positional relationship between the highest FB point and the FP, Matsui et al. reported that the highest FB point was on the nasal side of the FP in 97 eyes (66%) and the superior side of the FP in 82 eyes (55%) in a study of young, healthy, Japanese participants^17^. Though neither our study nor that of Matsui et al. studied the positional relationship between FP and the center of the FAZ, the report of Matsui et al. implies that FP is located inferior side of FB, and our results indicate that center of the FAZ is located inferior side of FB. Regarding positional relationship between highest FB point and center of the FAZ, Kuppuswamy et al. reported that the position of the FB nasally deviates from the FP in healthy eyes, with a median distance of 58.6 μm^22^. The results of the Kuppuswamy’s study were consistent with the results of our study regarding the position of the highest FB point in relation to the center of the FAZ. However, this study found no significant difference between the position of the highest FB point and the center of the FAZ in the horizontal direction. Hasegawa et al. reported the degree of misalignment between the center of the FAZ and the highest FB point in individuals with ERM in one eye and a healthy contralateral eye^16^. The mean distance from the highest FB point to the center of the FAZ was 38.7 ± 20.0 µm in the healthy eyes, which is consistent with the results of our study.

In the study of diseased eyes, a recent study by Hasegawa et al. reported that foveal misalignment was greater in eyes with ERM with ectopic inner foveal layers (EIFLs) than in those without EIFLs, especially in the horizontal direction, with an average distance of more than 100 μm^16^. The Hasegawa’s study reported that the degree of misalignment between the center of the FAZ and the highest FB point was greater in eyes with more advanced ERM, which may be useful for evaluating the degree of ERM traction. In our present study, eyes with a total distance of more than 50 μm between the center of the FAZ and the highest FB point had significantly greater misalignment in the horizontal direction than in the vertical direction. As the participants in this study were elderly individuals with healthy eyes, sub-OCTA structural changes may have occurred or precede in the eyes with greater foveal misalignment. Although these relationships are unclear due to the limitations of cross-sectional studies, the pathogenesis and progression of ERM should be studied in longitudinal studies in the future. Also, to clarify the pathophysiology of retinal diseases, further studies are needed to investigate the location of the center of FAZ and the highest FB point in various retinal diseases such as rhegmatogenous retinal detachment and retinal vein occlusion.

This study has several limitations. First, the age distribution of the participants in this study was not sufficiently wide, and the number of participants was not large. A previous report suggested that FB was more clearly observed in younger individuals and its height decreased with age^23^. However, age is not a specific confounder of this study as it compares the distance between the OCTA-derived center of the FAZ and the highest FB point according to the imaging setting in the same participant and does not compare the misalignment between age groups. Although there are no indications that this observation will not apply to individuals of different ages, future studies to confirm the distance in younger individuals are needed. Second, although the center of the FAZ was more likely to be located inferior to the highest FB point and was significantly different compared to a reference value of ± 0, the difference was relatively small (10 μm). Thus, even though the results showed statistical significance, they may not be clinically meaningful. However, the purpose of this study was to investigate the distance between the center of the automatically-determined, OCTA-derived FAZ area and the manually-determined highest FB point. The results of this study indicate that the center of the FAZ and the highest FB point are near one another in healthy individuals. These results will improve the understanding of diseased and treated eyes.

In conclusion, the distance between the center of the automatically-determined, OCTA-derived FAZ area and manually-determined highest FB point is minimal, but was not aligned in elderly participants with healthy eyes. These results can be applicable to further studies regarding the spatial relationships between the center of the FAZ and the highest FB point in various macular diseases or previously-treated eyes.

## Data Availability

The datasets generated and/or analyzed during the current study are available from the corresponding author upon reasonable request.

## References

[1] Bringmann A (2018). The primate fovea: Structure, function and development. Prog. Retin. Eye Res..

[2] Massin P (2002). Retinal thickness in healthy and diabetic subjects measured using optical coherence tomography mapping software. Eur. J. Ophthalmol..

[3] Drexler W, Fujimoto JG (2008). State-of-the-art retinal optical coherence tomography. Prog. Retin. Eye Res..

[4] Geitzenauer W, Hitzenberger CK, Schmidt-Erfurth UM (2011). Retinal optical coherence tomography: Past, present and future perspectives. Br. J. Ophthalmol..

[5] Hasegawa T, Ueda T, Okamoto M, Ogata N (2014). Relationship between presence of foveal bulge in optical coherence tomographic images and visual acuity after rhegmatogenous retinal detachment repair. Retina.

[6] Hasegawa T, Ueda T, Okamoto M, Ogata N (2014). Presence of foveal bulge in optical coherence tomographic images in eyes with macular edema associated with branch retinal vein occlusion. Am. J. Ophthalmol..

[7] Watanabe K, Tsunoda K, Mizuno Y, Akiyama K, Noda T (2013). Outer retinal morphology and visual function in patients with idiopathic epiretinal membrane. JAMA Ophthalmol..

[8] Chen CJ (2012). Characterizing the phenotype and genotype of a family with occult macular dystrophy. Arch. Ophthalmol..

[9] Al-Haddad CE, El Mollayess GM, Mahfoud ZR, Jaafar DF, Bashshur ZF (2013). Macular ultrastructural features in amblyopia using high-definition optical coherence tomography. Br. J. Ophthalmol..

[10] Chui TY, VanNasdale DA, Elsner AE, Burns SA (2014). The association between the foveal avascular zone and retinal thickness. Investig. Ophthalmol. Vis. Sci..

[11] Tang FY (2017). Determinants of quantitative optical coherence tomography angiography metrics in patients with diabetes. Sci. Rep..

[12] Koulisis N (2017). Quantitative microvascular analysis of retinal venous occlusions by spectral domain optical coherence tomography angiography. PLoS ONE.

[13] Choi J (2017). Quantitative optical coherence tomography angiography of macular vascular structure and foveal avascular zone in glaucoma. PLoS ONE.

[14] Kwon J, Choi J, Shin JW, Lee J, Kook MS (2017). Glaucoma diagnostic capabilities of foveal avascular zone parameters using optical coherence tomography angiography according to visual field defect location. J. Glaucoma.

[15] Shoji T (2020). OCT angiography measured changes in the foveal avascular zone area after glaucoma surgery. Br. J. Ophthalmol..

[16] Hasegawa T, Kawaguchi A, Arakawa H, Maruko I, Iida T (2020). Misalignment between center of foveal avascular zone and center of foveal photoreceptors in eyes with idiopathic epiretinal membrane. Retina.

[17] Matsui Y, Miyata R, Uchiyama E, Matsubara H, Kondo M (2020). Misalignment of foveal pit and foveal bulge determined by ultrahigh-resolution SD-OCT in normal eyes. Graefes Arch. Clin. Exp. Ophthalmol..

[18] Ishii H (2019). Automated measurement of the foveal avascular zone in swept-source optical coherence tomography angiography images. Transl. Vis. Sci. Technol..

[19] Zhang Q (2018). A novel strategy for quantifying choriocapillaris flow voids using swept-source OCT angiography. Investig. Ophthalmol. Vis. Sci..

[20] Bojikian KD (2016). Optic disc perfusion in primary open angle and normal tension glaucoma eyes using optical coherence tomography-based microangiography. PLoS ONE.

[21] Moghimi S (2012). Measurement of optic disc size and rim area with spectral-domain OCT and scanning laser ophthalmoscopy. Investig. Ophthalmol. Vis. Sci..

[22] Kuppuswamy Parthasarathy M, Bhende M (2018). Deviation in the position of foveal bulge from foveal center in normal subjects measured using spectral-domain OCT. Ophthalmol. Retina.

[23] Saurabh K (2017). Age-related changes in the foveal bulge in healthy eyes. Middle East Afr. J. Ophthalmol..

